# A case report of near-missed heat stroke

**DOI:** 10.1097/MD.0000000000036676

**Published:** 2023-12-22

**Authors:** Cheng Wooi Yeoh, Wan Chung Law

**Affiliations:** a Department of Medicine, Faculty of Medicine and Health Sciences, University of Malaysia Sarawak, Jalan Datuk Mohammad Musa, Kota Samarahan, Sarawak, Malaysia; b Department of Medicine, Sarawak General Hospital, Jalan Hospital, Kuching, Sarawak, Malaysia.

**Keywords:** coma, creatine kinase, heat stroke, heat-related illness, rhabdomyolysis, unconsciousness

## Abstract

**Rationale:**

Heat-related illnesses have protean manifestations that can mimic other life-threatening conditions. The diagnosis of heat stroke requires a high index of suspicion if the patient has been exposed to a high-temperature environment. Central nervous system dysfunction is a cardinal feature. Strict adherence to temperature criteria can potentially lead to misdiagnosis.

**Patient concerns:**

A 37-year-old construction worker was brought in by his wife and coworker due to a sudden loss of consciousness while resting after completing his work.

**Diagnoses:**

Due to challenges faced during the coronavirus disease 2019 pandemic, as well as language barriers, a detailed history from the coworker who witnessed the patient’s altered sensorium was not available. He was initially suspected of having encephalitis and brainstem stroke. However, subsequent investigations revealed multiorgan dysfunction with a normal brain computed tomography and cerebral computed tomography angiogram. In view of the multiple risk factors for heat stroke, pupillary constriction, and urine color suggestive of rhabdomyolysis, a diagnosis of heat stroke was made.

**Interventions:**

Despite delayed diagnosis, the patient’s multiorgan dysfunction recovered within days with basic supportive care.

**Outcomes:**

There were no noticeable complications on follow-up 14 months later.

**Lessons:**

Heat stroke can be easily confused with other neurological pathologies, particularly if no history can be obtained from the patient or informant. When approaching a comatose patient, we propose that serum creatinine kinase should be considered as an initial biochemical screening test.

## 1. Introduction

Heat-related illnesses consist of a spectrum of clinical conditions of varying severity. The heat stroke is at the extreme end of the continuum. Heat stroke is defined as a core body temperature of more than 40 ^0^C associated with central nervous system dysfunction.^[1]^ The temperature criteria was arbitrarily determined, and different studies in the past years used different cutoff values.^[2]^ Clinicians should recognize that the core body temperature of heat stroke casualties may vary with the timing of measurement or after cooling measures have been attempted. Furthermore, it is not a routine practice to measure rectal temperature without specific indications.

Heat stroke is a medical emergency. Prompt diagnosis, rapid cooling, and efficient supportive care are important elements in reducing morbidity and mortality.^[3,4]^ Most authors recommend cooling measures to be applied first in the field for heat stroke patients, then only transfer to the hospital.^[3,5]^

## 2. Case description

In January 2022, a 37-year-old male construction worker suddenly lost consciousness at his workplace at around 3.30 PM The day before, he had headache, back pain, and generalized weakness, but no fever. On the morning of the incident, he experienced a premonitory feeling of vague, indescribable discomfort. However, he still went to work. At noon, he experienced dizziness, but continued his piling work. He suddenly fell unconscious while resting after finishing his work for the day. His coworkers noted that he had a seizure. He was brought to a nearby clinic but was advised to visit the hospital. Due to the fear of contracting coronavirus disease 2019 (COVID-19), the coworker decided to send him back to his quarters and call for an ambulance. A wet towel was applied to his forehead while waiting for the ambulance.

**Figure 1.**
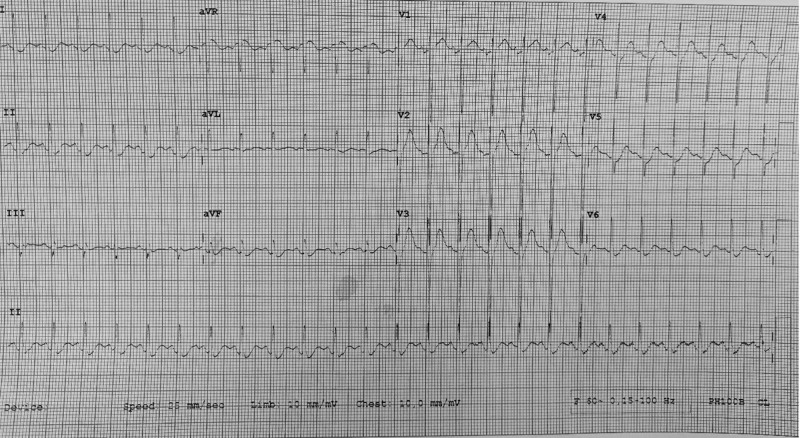
ECG on arrival at emergency department. (GRAYSCALE). ECG = electrocardiography.

During the active waves of the COVID-19 pandemic, our hospital implemented an infection control policy that permitted only one person to accompany the patient at any point in time. The wife was the person who accompanied him. On arrival at the hospital, the patient was restless and uncooperative. His blood pressure was 132/82 mm Hg, pulse rate was 154 beats per minute, temperature was 36.8 ^0^C (taken from scanning forehead by an infrared thermometer). Figure 1 shows the electrocardiography on arrival at the Emergency Department. He could move all his limbs. The patient was empirically treated for encephalitis by the attending team of doctors with intravenous Cephalosporin. He received intravenous diazepam 10 mg at 5.00 PM. An urgent plain computed tomography scan of the brain did not show any significant abnormalities. Due to his delirious state, the attending doctor administered intravenous midazolam 2 mg, followed by another intramuscular midazolam 2 mg, together with a dose of intramuscular haloperidol 5 mg at 7.25 PM. In view of the limited Intensive Care Unit bed and his stable condition, he was admitted to a General Medical Ward. The on-call team that reviewed him subsequently suspected the patient had a brainstem stroke.

During our review, his wife’s history revealed that he had been working as a construction worker for many years and had recently restarted his job 3 days prior, after being unemployed for more than 6 months during the COVID-19 pandemic. Usually, he wore a hat and 2 layers of clothing. His face fully covered with only the eyes exposed. The working hours were 6.00 am to 3.00 PM.

Upon examination, his Glasgow Coma Scale (GCS) score was 9/15 (eye - 2; verbal - 1; and motor - 5). The pupils were 2 mm and equal. The neck was supple. The patient had bilateral flaccid limbs and flexor plantar responses. His urine was orange-brown in color. We suspected severe heat-related illness and immediately performed a serum creatine kinase assay. His intravenous hydration was optimized. Cooling measures, including the removal of clothing and fanning were applied. The patient was then transferred to a ward with air conditioning several hours later. His GCS improved after 4 hours but was only able to tell his name. The brain magnetic resonance imaging scan was canceled.

His sensorium returned to normal, and pupillary constriction resolved more than 36 hours after the last dose of parenteral benzodiazepine. There was retrograde amnesia. He was able to ambulate with support on day 4 of admission. His body temperature was normal throughout the hospital stay. He was discharged on day 7 of admission and required another week to regain full muscle strength. Table 1 shows the serial investigation results during admission, which depict the affected organ systems.

**Table 1:** Serial investigation results.

Investigation	On admission	Day 2	Day 3	Day 4	Day 5	Day 6	Day 7	Reference range
Hemoglobin	14.6	14.4	12.9	12	11.8	15.2	13.9	12.5–16.5 g/dL
Total white cell	17.9	14.78	15.06	4.92	8.52	11.14	10.79	4–11 × 10^3^/μL
Platelet	244	89	58	53	83	191	158	142–424 × 10^3^/μL
PT	12.5	15.3	19.9			13.3		10.1–12.6 s
APTT	33.8	30.5	31.2			30		28.2–39.8 s
Sodium	141	140	137	138	136	137	137	135–145 mmol/L
Urea	8.6	12	7.2	3.81	3.4	3.3	3.5	1.7–8.3 mmol/L
Creatinine	255	242	108	76	67	80	63	60–120 µmol/L
Total bilirubin	14.4	13	22.2	56.2	34.8	31.1	20.4	3–18 µmol/L
AST		186	678	939	484	377	197	1–31 U/L
ALT	69	109	491	1494	1067	977	691	1–31 U/L
Albumin	38	30	28	28	26		29	35–52 g/L
Calcium	2.42	2.27	1.97					2.20–2.55 mmol/L
Phosphate	0.25	0.95	0.87					0.66–0.99 mmol/L
Creatine kinase		10,566	14,334		9589		4723	39–108 U/L
CK-MB		149						<25 U/L
Troponin T			96					<10 ng/L

Other investigation results: Venous blood gas (on arrival): pH: 7.322; pO_2_:44.7 mm Hg; pCO_2_: 33.8 mm Hg; HCO_3_^-^: 17.1; base excess: -7.9 mmol/L. Urine toxicology screen (post-IV diazepam): benzodiazepine detected. Peripheral blood film: no hemolysis, no platelet clump/fibrin seen. Blood culture: no growth. Blood film for malaria parasite: negative in three consecutive samples. Leptospira IgM: negative. Dengue serology: NS1 antigen/Ig M: negative; Ig G: positive. CT cerebral angiogram: no evidence of large vessel occlusion.

ALT = alanine aminotransferase, APTT = activated partial thromboplastin time, AST = aspartate aminotransferase, CK-MB = creatine kinase-myocardial band, CT = computed tomography, HCO_3-_ = bicarbonate, IgG = immunoglobulin G, IgM = immunoglobulin M, NS1 = nonstructural protein 1, pCO_2_ = partial pressure of carbon dioxide, pO_2_ = partial pressure of oxygen oxygen, PT = prothrombin time.

The patient was followed up until 14 months later. He was able to do farming work and return to usual self.

## 3. Discussion

Many conditions may present with high body temperatures and central nervous system dysfunction. Although our patient had a normal body temperature, the differential diagnoses of encephalitis or encephalopathy, brainstem stroke, cerebral malaria, severe dengue fever, thrombotic thrombocytopenic purpura, severe sepsis and drug intoxication had been considered.^[1]^ Anyway, subsequent investigations did not support the alternative diagnosis. Early recognition of heat-related illnesses could prevent unnecessary and costly investigations. As illustrated in this case, brain magnetic resonance imaging was called off upon diagnosis.

The patient was admitted during the COVID-19 pandemic whereby strict infection control measures were implemented. During this period, the noncontact infrared forehead temperature was recorded on arrival at the emergency department and throughout the hospital stay. No rectal temperature measurements were performed. However, an infrared thermometer is not reliable for assessing body temperature.^[6]^ Moreover, 2 hours had passed since his symptom onset until he had a documented temperature upon arrival at the Emergency Department. Rigid adherence to the temperature cutoff value without considering factors that could have affected the body temperature would lead to missing the diagnosis of heat stroke.^[4]^

Our patient had several predisposing factors for exertional heat stroke. Borneo has a tropical rainforest climate, characterized by high temperatures and high humidity. His job as a construction worker has subjected him to prolonged exposure to a high-temperature environment. Besides, the lack of acclimatization following a 6-month cessation of the job of a similar nature made him susceptible to heat-related illness. Furthermore, his thick clothing may protect him from direct sunlight, but limits heat dissipation via convection and sweat evaporation.^[7]^

Exertional heat stroke has a lower mortality rate than classic heat stroke.^[3,4]^ However, the actual mortality rate in the general population is unknown, mainly because of misdiagnosis and incomplete data. The reported mortality rate of exertional heat illness varies widely between studies, ranging from 0% to 33%.^[1,8]^ Although the majority of the patients survived the acute episode of heat stroke, some survivors developed long-term neurologic and renal complications.^[9]^ Fortunately, our patient recovered without lasting sequelae despite delayed diagnosis, and no immediate cooling attempt was performed in the field. It is possible that both general supportive care and individual factors play important roles in the prognosis of severe heat-related illnesses.^[10]^ It is likely that timely removal of this patient from the environment and management of his delirious state with sedative drugs stopped further exertion, thereby reducing further heat production. In addition, the Emergency Department was air conditioned. Changing his clothing to thin hospital attire and intravenous hydration promoted heat dissipation.

It is well recognized that pupillary constriction can occur in heat stroke.^[11]^ In fact, this patient’s constricted pupils without upper motor neuron signs or cranial nerve palsies led to the suspicion of heat stroke upon transfer under our care. Intuitively, his pupillary constriction may be attributable to the administration of multiple doses of parenteral benzodiazepines (10 mg diazepam and 4 mg midazolam in total) at the Emergency Department. However, the pupil constriction lasted more than 36 hours. Midazolam has a short half-life. On the other hand, the sedative effect of diazepam is not associated with pupil constriction.^[12]^ Nevertheless, it is uncertain whether transient acute liver dysfunction has any effect on benzodiazepine metabolism.

Most of the patient’s history was obtained only after regaining consciousness. Additional details were gathered from a coworker who had witnessed his collapse. During admission, only a limited history could be obtained from the wife, as she was the only informant available. His wife spoke local dialect and slang, which contributed to a communication barrier for us.

We only managed to contact the patient’s coworker, who witnessed his sudden loss of consciousness during that day, more than one year after his discharge. According to the coworker, he saw the patient suddenly fell down while sitting in a pavilion. Upon approaching him, the patient was drooling bubbly saliva and the limbs were stiff and jerking. He was unresponsive with the eyes closed. There was no urinary incontinence or tongue biting noted. This description suggested that the patient had a generalized tonic-clonic seizure.

Assessment of patients who are unable to provide a history can be a challenging task for clinicians. In a culturally diverse society and with the increasing popularity of international travel, clinicians may occasionally encounter patients with altered sensorium or language barriers. This could pose a diagnostic challenge, especially when a reliable informant or interpreter is unavailable. The importance of information about the timing, condition of the patient, and environment in which the patient was found could not be overemphasized. Careful systematic and targeted clinical examinations from head to toe are essential.^[13,14]^ In view of the broad differential diagnoses for unconsciousness, some basic initial screening blood investigations may be useful to guide further investigation or management.

**Figure 2.**
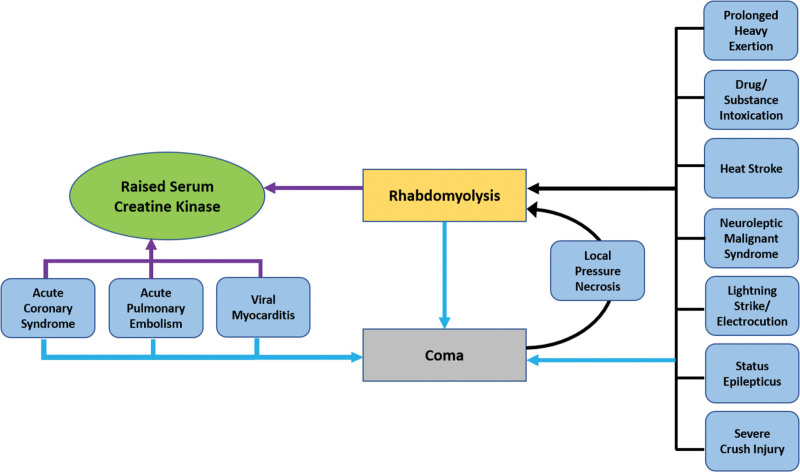
Conditions that may present with coma and associated with raised serum creatine kinase. (RGB); Black arrows: conditions that may lead to rhabdomyolysis; blue arrows: conditions that can present as coma; purple arrows: conditions that can manifest as raised serum creatine kinase.

Rhabdomyolysis can result from prolonged coma; conversely, certain conditions that can cause rhabdomyolysis may result in coma.^[13–15]^ Thus, we suggest that serum creatine kinase should be included as part of the initial workup for acutely unconscious patients when a reliable history cannot be obtained from the patient or any informant. To illustrate the relationship between elevated serum creatine kinase levels and coma, we created a diagram, as shown in Figure 2. Although creatinine kinase is not a specific biomarker, it is relatively cheap and readily accessible in resource-limited settings. It can be used to screen for many conditions associated with an altered sensorium.

## 4. Patient perspective

In retrospect, the patient felt that the degree of exertion was not particularly high before he lost consciousness. It took him by surprise upon regaining consciousness in the hospital. He found that physiotherapy was helpful in regaining muscle strength.

## Acknowledgments

We are indebted to Dr Huong Nai Law for reviewing and editing the manuscript. We would like to thank the University of Malaysia Sarawak for providing the necessary support for this publication. The authors would also like to thank the patient and Director General of Health, Ministry of Health, Malaysia, for permitting access to the patient’s medical records for publication.

## Author contributions

**Conceptualization:** Cheng Wooi Yeoh.

**Data curation:** Cheng Wooi Yeoh.

**Formal analysis:** Cheng Wooi Yeoh, Wan Chung Law.

**Investigation:** Cheng Wooi Yeoh, Wan Chung Law.

**Software:** Cheng Wooi Yeoh.

**Supervision:** Cheng Wooi Yeoh, Wan Chung Law.

**Validation:** Cheng Wooi Yeoh, Wan Chung Law.

**Visualization:** Cheng Wooi Yeoh.

**Writing – original draft:** Cheng Wooi Yeoh.

**Writing – review & editing:** Cheng Wooi Yeoh, Wan Chung Law.
